# Endothelial Dysfunction and Atherosclerosis in Patients With Autosomal Dominant Polycystic Kidney Disease

**DOI:** 10.7759/cureus.13561

**Published:** 2021-02-25

**Authors:** İskender Ekinci, Mitat Buyukkaba, Ahmet Cinar, Muhammed Tunc, Egemen Cebeci, Meltem Gursu, Rumeyza Kazancioglu

**Affiliations:** 1 Department of Internal Medicine, SBU Istanbul Kanuni Sultan Süleyman Training and Research Hospital, Istanbul, TUR; 2 Department of Internal Medicine, Kanuni Sultan Suleyman Training and Research Hospital, Istanbul, TUR; 3 Department of Internal Medicine, Arnavutkoy State Hospital, Istanbul, TUR; 4 Department of Nephrology, Bezmialem Foundation University, Istanbul, TUR; 5 Department of Nephrology, Haseki Training and Research Hospital, Istanbul, TUR

**Keywords:** endothelial dysfunction, endocan, asymmetric dimethylarginine, autosomal-dominant polycystic kidney disease, flow mediated dilatation, nitroglycerin mediated dilation, carotid intima-media thickness, atherosclerosis

## Abstract

Introduction

In this study, we aimed to determine the endothelial dysfunction (ED) and atherosclerosis in patients with autosomal dominant polycystic kidney disease (ADPKD).

Materials and methods

This study was conducted with 83 subjects (26 male, mean age: 46±11 years) consisted of three groups including ADPKD, hypertension (HT) and healthy control groups. The groups were evaluated in terms of serum endocan and asymmetric dimethylarginine (ADMA) levels, flow-mediated dilatation (FMD), nitroglycerin-mediated dilation (NMD) and carotid intima-media thickness (CIMT).

Results

Serum endocan and ADMA levels and CIMT were significantly higher while NMD was significantly lower in ADPKD group than control group. FMD and NMD were lower but serum ADMA level was higher in the ADPKD group than HT group; while serum endocan level and CIMT were not significantly different in ADPKD and HT groups. In ADPKD patients, CIMT value and serum endocan and ADMA levels were higher while NMD was lower in patients with eGFR≤60 mL/min/1.73 m^2^ than patients with eGFR>60 mL/min/1.73 m^2^. Serum ADMA level was higher and NMD was lower in hypertensive ADPKD patients than non-hypertensive ones. Serum endocan level was higher in ADPKD patients with nephrolithiasis and a negative correlation was detected between serum endocan level and 24-hour urine volume.

Conclusions

Endothelial dysfunction and atherosclerosis are common conditions in ADPKD patients and it was further reinforced in our study. In order to clarify the relationship between serum endocan level and 24-hour urine volume, which is a remarkable finding in our study, larger studies that including the measurement of urine endocan may be useful.

## Introduction

Autosomal dominant polycystic kidney disease (ADPKD) is the most common hereditary kidney disease with a prevalence of 1/400-1000 and responsible for approximately 10% of end-stage renal disease [1]. The disease is characterized by the formation of epithelial cell cysts, enlargement in the extracellular matrix, and vascular alterations due to compression by the cysts [1]. Cardiovascular manifestations are common and start very early during the course of the disease and the main cause of morbidity and mortality in patients with ADPKD, where hypertension (HT) and endothelial dysfunction (ED) are among the most important contributors to this risk [2,3].

Endocan, previously called endothelial cell-specific molecule-1, is a soluble proteoglycan secreted by vascular endothelial cells and thought to play a role in the pathogenesis of ED and inflammation [4]. The secretion of endocan is increased in many endothelium-dependent pathological states such as inflammation, sepsis, malignancies, hypertension and atherosclerosis. High serum endocan levels and its correlation with other inflammation and ED-related parameters were reported in chronic kidney disease (CKD), acute kidney injury, peritoneal dialysis and transplanted patients [5-8].

Nitric oxide (NO) is an endothelium-derived molecule with a dilating effect on smooth muscle and is synthesized by nitric oxide synthase (NOS). Asymmetric dimethylarginine (ADMA) is an endogenous inhibitor of NOS and reduces NO level. ADMA has been reported to be elevated in patients with CKD and to be associated with endothelial dysfunction, atherosclerosis and cardiovascular disease [9,10].

Measurement of the flow-mediated dilatation (FMD) and nitroglycerin-mediated dilation (NMD) of brachial arteries and intima-media thickness of carotid arteries (CIMT) are the frequently used non-invasive methods for the determination of ED and atherosclerosis. While impaired FMD and NMD indicate the smooth muscle cell dysfunction and ED, an increased level of CIMT predicts the generalized atherosclerosis and cardiovascular events. Increased value of CIMT and impaired FMD and NMD have been reported previously in CKD patients [11-13].

In this study, we aimed to investigate the ED and atherosclerosis in ADPKD patients through serum endocan and serum ADMA levels and FMD, NMD and CIMT.

## Materials and methods

Patients and study design

The study was conducted with three groups: the first group consisted of ADPKD patients, the second group consisted of essential hypertension patients and the third group consisted of healthy volunteers. All subjects were over 18 years old. The participants for ADPKD and HT groups were selected from patients who were followed up in the nephrology outpatient clinic. The healthy subjects in the control group were selected from among those who applied to the outpatient clinic for routine (check-up) examination and have no disease. The diagnosis of ADPKD was based on sonographic criteria as described by Ravine et al. [14]. The diagnosis of hypertension was established per the European Society of Hypertension and the European Society of Cardiology ESH-ESC 2013 guidelines for hypertension [15].

Exclusion criteria

Patients with renal replacement therapies, type 1 and type 2 diabetes mellitus, acute renal failure, a history of malignancy, active infection, chronic liver disease, advanced cardiac disease, inflammatory diseases, psoriasis, chronic lung diseases (chronic obstructive pulmonary disease, bronchiectasis, asthma and pulmonary hypertension), a history of cerebrovascular disease, a history of thyroid pathology, morbid obesity, Alzheimer's disease or dementia, pregnancy and a history of recently performed heavy exercise were excluded from the study. Subjects younger than 18 years were excluded from the study while no upper age limit was considered as an exclusion criterion.

Patient assessments

A detailed physical examination was performed on all study subjects. Data regarding disease-related conditions (presence of hematuria, nephrolithiasis, liver cyst and mitral valve prolapsed [MVP]) of ADPKD patients were collected from medical records. The duration elapsed after the diagnosis of ADPKD, presence of HT and smoking status were recorded. The systolic and diastolic blood pressure (SBP and DBP) measurements were performed on the right arm with the use of a mechanic sphygmomanometer after 15 minutes of resting. Bodyweight, expressed as kilogram, was measured with a platform scale.

The following laboratory parameters were measured: serum endocan, serum ADMA, complete blood count, blood urea nitrogen (BUN), creatinine (Cr), uric acid (UA), calcium, phosphorus, sodium, potassium, hemoglobin (Hgb), 24-hour urine protein and 24-hour urine volume.

Blood sampling and blood analysis

Venous blood sampling was carried out in the early morning after overnight fasting. Blood samples obtained from patients were placed in gel tubes and a 20-minute interval was allowed for coagulation before centrifuging for 15 minutes at 1,500 G. Sera were stored at −80°C for endocan and ADMA assay, which was carried out in the whole group of samples at a later date. 

Complete blood count (CBC) analysis was performed using a Sysmex XT 1800i device (Roche-2011, Kobe, Japan). BUN, Cr, UA, sodium, potassium, calcium and phosphorus in the sera were analyzed using an Architect ci16200 (Abbott, Abbott Park, IL, USA) device.

Urine samples and urinalysis

24-hour urine was collected as previously described by Kouri et al. 16- to 24-hour urinary protein was measured turbidimetrically using an Architect CI 16200 (Abbott, USA) device.

Estimated glomerular filtration rate: eGFR was estimated by the abbreviated modification of diet in renal disease equation [16]:

eGFR (mL/min/1.73 m^2^) = 186.3 x SCr (exp[-1.154]) x Age (exp[-0.203]) x (0.742 if female) x (1.21 if black).

ELISA assay

Concentrations of endocan in serum were measured with enzyme-linked immunosorbent assay (ELISA) kit, according to protocols provided by manufacturers. Multiscan FC® Microplate Photometer (Thermo Fisher Scientific, Waltham, MA, USA) was used for reading at 450 nm. The results were expressed as pg/mL. Concentrations of ADMA in serum were measured with ELISA kit according to protocols provided by manufacturers. BioTekTM ELx 50 washer and ELx 800 reader were used for reading at 450 nm. The results were expressed as µmol/L.

Assessment of endothelial dysfunction and atherosclerosis: Evaluation of vascular endothelial function by FMD was performed according to a published protocol [17]. The participants were in a state of fasting for at least eight hours. All vasoactive medications were withheld for 24 hours before the procedure. The subjects remained at rest in the supine position for at least 15 minutes before the examination started. Each participant was examined in supine position with a blood pressure cuff placed around the right arm below the antecubital fossa. The brachial artery was scanned in the longitudinal section using a 12 MHz linear array transducer (GE Logic 9, Milwaukee, WI, USA). For FMD and NMD measurements, firstly brachial artery baseline diameter was obtained. For FMD measurement, brachial artery peak diameter was obtained after stretching the forearm for 5 min. After the release of the cuff, serial measurements were obtained in 1 min. Ten minutes after recovering FMD, sublingual isosorbid dinitrate 5 mg was administered and the diameter of the brachial artery was measured in 3 min. The mean value of five measurements was calculated for both FMD and NMD. The diameter of the brachial artery was measured coinciding with the R wave of the ECG.

FMD (%) = (Peak diameter - baseline diameter) / (baseline diameter)

NMD (%) = 100 X [(Diameter after administration of nitrate - baseline diameter) / (baseline diameter)]

CIMT measurements were obtained with the patient lying in the supine position and with the neck slightly rotated to the opposite side of the examination. The bulbus of the common carotid artery was scanned by longitudinal views using a 12 MHz linear array transducer (GE Logic 9, Milwaukee., WI, USA). At least three intima-media thickness points were measured in the near and far walls in the most thickened area of each vessel. The average of the three values was recorded as mm.

Statistical analysis

Analyses were performed using SPSS version 15.0 for Windows (IBM Corporation, Chicago, IL, USA). Categorical variables were expressed as numbers and percentages; numeric variables were expressed as mean ± standard deviation. Categorical data between more than two groups were obtained by use of the chi-square test. Shapiro-Wilk was used to detect whether the data were normally or non-normally distributed. For >2 numeric variables; ANOVA was used when the data were normally distributed and Kruskal-Wallis was used when non-normally distributed, which was interpreted after Bonferroni correction. For comparison of numeric variables between two groups, student’s t-test was applied when data were normally distributed and Mann-Whitney U-test when non-normally distributed. Pearson correlation test and Spearman’s correlation test were used to detect the association between parameters for the normally and non-normally distributed parameters, respectively. A p-value <0.05 was regarded as statistically significant.

Ethical considerations

The study protocol was approved by Bezmialem Foundation University Medical Faculty Ethics Committee (No: 71306642-050.01.04-), and all participants recruited to the study provided written informed consent. The study procedures were performed in accordance with the 2009 Helsinki Declaration.

Informed consent

Informed consent was obtained from all individual participants included in the study.

## Results

The study included 83 patients (57 females) and the mean age was 46±11 years (min-max: 20-68 years). Mean duration elapsed after diagnosis in ADPKD patients was 11.1±8 years. The frequency of clinical findings in patients with ADPKD was as follows: HT 62.7% hematuria 11.7%, nephrolithiasis 33.3%, liver cyst 13.7 % and MVP 37.2%.

Evaluation of subjects was based on three pre-determined subgroups: ADPKD group, HT group and the control group. The baseline characteristics and the laboratory parameters of the groups were presented in Table 1. The three groups were compatible in terms of age, gender, smoker status and mean weight. The eGFR value was significantly lower in the ADPKD group than HT and control groups. The highest blood pressure measurements among the groups were observed in the HT group. Serum creatinine level was higher in the ADPKD group than HT and control groups, uric acid level was higher in ADPKD group than the control group and Hgb value was lower in ADPKD group than control group. The level of 24-hour urine protein was higher in the ADPKD group than both HT and control groups.

**Table 1:** Demographic data and laboratory parameters of the subjects. ^*^HT group vs control group, p: 0.012; ^**^ADPKD group vs HT group, p: 0.007; ^***^HT group vs control group, p:<0.001; ^#^ADPKD group vs HT group; p: 0.014; ^##^ADPKD group vs control group, p: 0.003; ^£^ADPKD group vs HT group, p: 0.001; ^££^ADPKD group vs control group, p: 0.035; ^£££^ADPKD group vs control group, p: 0.024; ^$^ADPKD group vs control group, p: 0.034; ^$$^ADPKD group vs HT group, p: 0.007; ^$$$^ADPKD group vs control group, p: 0.001. Abbreviations: systolic blood pressure (SBP), diastolic blood pressure (DBP), estimated glomerular filtration rate (eGFR), blood urea nitrogen (BUN), creatinine (Cr), uric acid (UA), and hemoglobin (Hgb), blood urea nitrogen (BUN), creatinine (Cr), uric acid (UA), and hemoglobin (Hgb), autosomal dominant polycystic kidney disease (ADPKD), hypertension (HT).

	ADPKD group (n = 51)	HT group (n = 20)	Control group (n = 12)	p
Age	45.5±12.3	47.2±8.9	45.8±13	0.872
Gender, n (female/male)	34/17	17/3	6/6	0.104
Smoker, n (%)	14 (%27.5)	7 (%35)	7 (%58.3)	0.125
Weight (kg)	77.4±17.1	77.1±13.8	70.3±11.8	0.46
SBP (mmHg)	121.2±13.5	127.5±12.6	114.1±13.1	0.012^*^
DBP (mmHg)	70.5±7.4	75.7±4.9	64.5±7.5	<0.001^**, ***^
eGFR (ml/min/1.73 m^2^)	70.68±29.6	93.15±11.6	99.4±14.9	<0.001^#, ##^
BUN (mg/dL)	19.5±11.9	13.2±3.4	14.2±5.2	0.110
Cr (mg/dL)	1.28±1.09	0.72±0.07	0.76±0.74	0.001^£, ££^
UA (mg/dL)	5.7±1.8	4.8±1.3	4.5±1.2	0.028^£££^
Calcium (mg/dL)	9.7±0.4	9.7±0.3	9.4±0.2	0.110
Phosphorus (mg/dL)	3.5±0.4	3.4±0.5	3.5±0.5	0.791
Sodium (mg/dL)	139.6±2.3	139.4±2.1	139.2±2.1	0.868
Potassium (mg/dL)	4.3±0.3	4.2±0.2	4.3±0.3	0.601
Hgb (g/dL)	12.9±1.6	13.3±1.4	14.2±1.4	0.036^$^
urine protein (mg/day)	469.7±1007	156.5±67.4	130.3±55.3	<0.001^$, $$^
Urine volume (ml/day)	2527.8±862.1	2232±782	2556±800	0.378

The data relating to ED-associated parameters are presented in detail in Table 2. Serum endocan level was significantly higher in both ADPKD and HT group compared to control group but no significant difference was found between ADPKD and HT groups. Serum ADMA level was significantly higher in ADPKD group compared to each of HT and control group. CIMT was significantly higher in both ADPKD and HT group compared to control group. FMD was significantly lower in ADPKD group compared to HT group. NMD was significantly lower in ADPKD group compared to each of HT and control group.

**Table 2:** The evaluation of parameters related to endothelial dysfunction and atherosclerosis in the groups. ^*^ADPKD group vs HT group, p: 0.017; ^**^ADPKD group vs HT group, p: 0.048; ^***^ADPKD group vs control group, p: 0.016; ^#^ADPKD group vs HT group, p: <0.001; ^##^ADPKD group vs control group, p: 0.016; ^£^ADPKD group vs control group, p: 0.021; ^££^HT group vs control group, p: 0.002; ^$^ADPKD group vs control group, p: 0.001; ^$$^HT group vs control group, p: 0.001. Abbreviations: flow-mediated dilatation (FMD), nitroglycerin-mediated dilation (NMD), asymmetric dimethyl-arginine (ADMA), carotid intima-media thickness (CIMT), autosomal dominant polycystic kidney disease (ADPKD), hypertension (HT).

	ADPKD group	HT group	Control group	p
FMD, %	6.91±6.07	10.07±5.63	9.22±4.35	0.026^*^
NMD, %	20.25±10.62	26.86±11.43	22.61±3.83	0.05^**, ***^
ADMA, µmol/L	0.46±0.05	0.39±0.05	0.41±0.04	<0.001^#, ##^
CIMT, mm	0.8±0.26	0.83±0.24	0.63±0.12	0.043^£, ££^
Endocan, pg/ml	84.7±63.9	87.0±53.8	32.7±7.5	0.001^$, $$^

The analysis of the endothelial dysfunction and atherosclerosis associated parameters in patients with ADPKD while the ADPKD patients were grouped according to eGFR levels, presence of hypertension, nephrolithiasis and liver cyst are shown in Table 3.

**Table 3:** The analysis of endothelial dysfunction associated parameters in patients with ADPKD by subgroups. Abbreviations: estimated glomerular filtration rate (eGFR), flow-mediated dilatation (FMD), nitroglycerin-mediated dilation (NMD), asymmetric dimethyl-arginine (ADMA), carotid intima-media thickness (CIMT), autosomal dominant polycystic kidney disease (ADPKD).

	n	Endocan, pg/ml	FMD, %	NMD, %	ADMA, µmol/L	CIMT, mm
eGFR, ≤60 ml/min/1.73 m^2^	18	87±74.8	6.2±5	16.9±10	0.48±0.04	0.9±0.2
eGFR, >60 ml/min/1.73 m^2^	33	75.2±54.6	7.2±6	22±10.6	0.45±0.06	0.7±0.2
p		<0.001	0.608	0.021	0.038	0.023
Hypertension, yes	32	95.6±75	6.2±4.9	17.2±9.9	0.49±0.04	0.83±0.27
Hypertension, no	19	66.4±32.9	7.9±7.7	25.3±10	0.42±0.05	0.76±0.25
p		0.355	0.453	0.008	<0.001	0.283
Nephrolithiasis, yes	17	109.6±79	6.9±4.7	19.3±8.8	0.47±0.04	0.81±0.22
Nephrolithiasis, no	34	72.2±51.7	6.9±6.7	20.6±11.5	0.46±0.06	0.81±0.28
p		0.034	0.99	0.68	0.94	0.062
Liver cyst, yes	7	136.5±71.1	6.4±4.6	19.5±7.8	0.47±0.06	0.81±0.17
Liver cyst, no	44	76.5±59.4	6.9±6.3	20.3±11	0.46±0.05	0.80±0.27
p		0.019	0.978	0.681	0.341	0.941

Serum endocan and ADMA levels and CIMT value were higher while NMD was lower in ADPKD patients with eGFR ≤60 mL/min/1.73 m^2^ compared to eGFR >60 mL/min/1.73 m^2^ patients. While all ADPKD patients with eGFR ≤60 mL/min/1.73 m^2^ had HT, the rate of HT was 42% in ADPKD patients with eGFR>60 mL/min/1.73 m^2^.

No significant difference was found between hypertensive and non-hypertensive ADPKD patients in terms of serum endocan levels, FMD and CIMT. NMD was lower and ADMA was higher in hypertensive ones. The mean eGFR value was lower in hypertensive ADPKD patients compare to non-hypertensive patients (56.75±27.63 mL/min/1.73 m^2^ vs 94.15±14.15, p < 0.001).

Serum endocan levels were significantly higher in ADPKD patients with nephrolithiasis compared to those without nephrolithiasis while ED-related other parameters were similar between the two groups. 24-hour urine volume was lower in ADPKD patients with nephrolithiasis than those without nephrolithiasis (1985.8±576.2 ml vs 2798.8±147.3 ml; p: 0.001).

Serum endocan levels were significantly higher in ADPKD patients with liver cysts compared to those without liver cysts while ED-related other parameters were similar between the two groups.

Correlations

Correlation analysis of endothelial dysfunction and atherosclerosis-related parameters (serum endocan, serum ADMA, FMD, NMD and CIMT) with eGFR, creatinine, 24-hour urine volume and 24-hour urine protein level were analyzed. eGFR has a positive correlation with NMD (r: 0.326, p: 0.02) and a negative correlation with CIMT (r: -0.408, p: 0.003) and ADMA (r: -0.306, p: 0.029). CIMT has a positive correlation with creatinine (r: 0.331, p: 0.018) and 24-hour urine protein (r: 0.468, p: 0.001). Serum endocan has a negative correlation with 24-hour urine volume (r: -0.276, p: 0.05).

## Discussion

In this study, the presence of the ED and atherosclerosis in patients with ADPKD were observed clearly. The higher level of serum endocan, ADMA and CIMT value and the lower values of the FMD and NMD in ADPKD patients were the main findings of our study which support the hypothesis. The levels of ED-related parameters were detected more obvious in the ADPKD patients in the presence of low GFR and HT. In addition, the serum endocan levels were found to be higher in ADPKD patients with nephrolithiasis and liver cyst.

In ADPKD patients, the main causes of morbidity and mortality were cardiovascular problems, which were implied to develop secondary to atherosclerosis, ED and HT. Presence of ED and increased CIMT in young normotensive ADPKD patients with well-preserved renal functions suggests that atherosclerosis and ED develop early in the disease course [17-19]. Wang et al. reported that ADPKD patients with neither associated HT nor decreased renal function had impaired vascular relaxation and increased levels of ADMA, becoming more marked when HT developed in these patients which were associated with decreased NO synthase activity [13,20]. Serum ADMA levels have been found higher in ADPKD patients than in healthy subjects and an association between ADMA and eGFR but not with ADMA and creatinine was pointed in Klawitter et al study [21]. Kocaman et al. showed that FMD levels in ADPKD were lower than that in healthy controls yet similar to that in HT patients and this reduction became more marked in hypertensive ADPKD patients. In the same study, NMD was also regarded as significantly impaired in ADPKD and HT patients as compared to the control group [17]. In addition, before HT developed, CIMT was reported to be higher in ADPKD patients compared to healthy controls, which was more markedly increased after HT development [17]. Contrary to the literature low serum ADMA level and non-different CIMT values were stated in another study [22]. In our study, the rate of hypertensive patients in ADPKD group was found as higher as 63.7%, similar to those reported in the literature. We detected an elevated level of serum endocan and ADMA, increased CIMT and low levels of NMD and FMD as the main findings indicating ED.

Balta et al. reported elevation of endocan levels in HT patients and association of the endocan levels with CIMT and CRP [23]. Endocan levels were reported to rise with a concomitant reduction of eGFR in patients with chronic kidney disease, secondary to inflammation and vascular dysfunction, and predict all-cause mortality and cardiovascular events [5]. Serum endocan levels were found higher in CKD patients than healthy subjects and in addition that serum endocan levels were found to be higher in CKD patients with CVD as compared to those without CVD [6]. In another study, serum endocan levels were observed higher in peritoneal dialysis patients than healthy subjects [24]. Raptis et al. have reported higher serum endocan levels in ADPKD patients with eGFR 45-70 mL/min/1.73 m^2^ than ADPKD patients with eGFR >70 mL/min/1.73 m^2^ and higher serum endocan levels in ADPKD patients than controls [7]. Although having a similar renal function, serum endocan levels were found higher in ADPKD patients with eGFR >70 mL/min/1.73 m^2^ than healthy subjects in the same study. On the other hand, the serum endocan levels have also been reported to increase in patients with acute kidney injury [8]. Endocan levels were higher in both ADPKD and HT patients as compared to healthy controls in our study. Serum endocan levels were higher in ADPKD patients with eGFR ≤60 mL/min/1.73 m^2^ compared to ADPKD patients with eGFR above this value while no significant difference was found between hypertensive and non-hypertensive ADPKD patients. No influence on endocan levels by either the presence or absence of HT in ADPKD patients may be explained by the fact that most hypertensive ADPKD patients had controlled hypertension. We can express this by the fact that SBP and DBP averages are not different between the ADPKD group and the control group. We base this hypothesis on the fact that SBP and DBP mean values were not determined differently between the ADPKD group and the control group in our study. Another possible reason for this indifference may be that most patients have HT from the early stages of the disease.

ADPKD patients were reported to have an increased number of renal cysts, reduced creatinine clearance and 24-hour urine volume in the presence of nephrolithiasis [25]. Consistently, compared to ADPKD patients having no hepatic cysts, those with hepatic cysts were shown to have increased total kidney volume, total number of cysts and larger size of predominant cysts as well as reduced creatinine clearance [26]. In our study, we found higher serum endocan levels and lower 24-hour urine volume in ADPKD patients while disease accompanied with nephrolithiasis and liver cysts. Both elevation of serum endocan levels and decrease of 24-hour urine volume in ADPKD patients with nephrolithiasis may be associated with reduced urinary excretion or increased endocan secretion in response to endothelial injury. Significant negative correlation between the 24-hour urine volume and serum endocan levels considered as a supporting finding of the relationship between two parameters. Additional studies where urinary endocan levels will be assessed are required to make further accurate and comprehensive comments on this subject.

Limitations of our study include small number of samples, unmeasured urine endocan level and consecutive serum endocan measurements. Small sample size in our study warrants our findings to be supported by larger studies. Absence of the total kidney volume data was another limitation of this study.

## Conclusions

In conclusion, endothelial dysfunction and atherosclerosis are common conditions in ADPKD patients and it was further reinforced in our study too. To clarify the relationship between serum endocan level and 24-hour urine volume, new studies that including the measurement of urine endocan may be useful.
